# Intersectional inequalities in health anxiety: multilevel analysis of individual heterogeneity and discriminatory accuracy in the SOMA.SOC study

**DOI:** 10.3389/fpubh.2024.1388773

**Published:** 2024-06-26

**Authors:** Rieke Barbek, Daniel Lüdecke, Olaf von dem Knesebeck

**Affiliations:** Institute of Medical Sociology, University Medical Center Hamburg-Eppendorf, Hamburg, Germany

**Keywords:** health anxiety, intersectionality, MAIHDA, mental health disparities, social determinants of health, SOMA.SOC

## Abstract

**Background:**

Intersectional approaches are needed to disaggregate the complex interaction of social identities contributing to (mental) health disparities. Health anxiety represents an overlooked public mental health issue. Therefore, intersectional inequalities in health anxiety were examined using multilevel analysis of individual heterogeneity and discriminatory accuracy (MAIHDA).

**Methods:**

Analyses are based on cross-sectional data of the adult population living in Germany (*N* = 2,413). Health anxiety was assessed with the Whiteley Index-7. Applying intersectional MAIHDA, health anxiety in the intersectional strata of gender, history of migration, and income was predicted. Discriminatory accuracy was assessed via the intra-class correlation and the proportional change in variance.

**Results:**

Analyses revealed additive social inequalities in health anxiety with greatest impact of low income but no clear intersectional gradient. Most affected by health anxiety were females who immigrated themselves with low income, males whose parent(s) immigrated with low income, and males who immigrated themselves with medium income.

**Conclusion:**

Intersectional approaches contribute to a more comprehensive understanding of (mental) health disparities. In addition to general efforts to counteract health inequalities, combining universal screening and targeted psychotherapeutic treatment seems promising to specifically reduce inequalities in health anxiety.

## Introduction

Health anxiety is understood as the excessive worry over health and fear of having or getting a serious illness which can range from mild symptoms to a pathological extent (1, 2). With prevalence rates between 2.1 and 13.1% in the general population and even up to 34.6% in the psychiatric setting, health anxiety is a widespread but much overlooked challenge (1, 3). Due to its stress relation, health anxiety often coexists with other mental disorders, such as generalized anxiety disorder, depression or somatoform disorders and is also relevant in the context of (chronic) physical illnesses (4, 5). Adverse life events, e.g., experiencing a (former) serious illness, high levels of stress, or ongoing challenges in life can contribute to the development or exacerbation of health anxiety symptoms (4, 5). Health anxiety is associated with reduced health-related quality of life and high economic burden due to inappropriate health care use, sick-leave, and disability (1, 4, 6). Cognitive behavioral therapy has proven to be an effective treatment to reduce health anxiety (7). The challenge, however, remains to sensitize those affected to their excessive fears and enable access to appropriate psychotherapeutic treatment (7). In medical settings, brief screening instruments like the Whiteley Index (WI) or the Short Health Anxiety Inventory (SHAI) seem promising to identify individuals critically affected by health anxiety (1). This combination of universal screening and targeted treatment follows the approach of proportionate universal interventions (8).

It is important to acknowledge that social inequalities can play a role in shaping the experiences and outcomes of individuals with health anxiety. Systematic evidence revealed individuals with low socio-economic status, education, or income to be more affected by health anxiety (1, 9). However, regarding gender, age, and ethnic minority status/history of migration as further important social determinants of mental health (10), systematic evidence is inconclusive (1, 11). As former studies are limited to single indicators, an intersectional approach is needed to shed further light on the social context of health anxiety as an important and overlooked mental health outcome (3).

Intersectionality is rooted in Black feminist theory and can be understood as a framework to understand interactions of multiple social categories leading to disadvantage, discrimination, and inequality (12, 13). Stress exposure due to, e.g., sexism or racism and subsequent coping mechanisms can result in higher morbidity and mortality (14). Besides experiences of discrimination on the individual level, multiple discrimination can occur on a structural level diminishing peoples’ social power and opportunity for political participation (15). Additionally, protective factors to resist against one’s social categories might moderate the effect of intersections on health outcomes (16). Thereby, social mechanisms can occur either additive or multiplicative or some combination of both. Additive effects represent the sum of privileges and/or disadvantages, whereas multiplicative effects imply that the effects of the social characteristics enhance each other (16, 17). Quantitative research on intersectional inequalities in different health outcomes received increased attention in recent years and new statistical approaches have been developed (18–20). Research mainly focused on self-rated physical and/or mental health status as outcome and on the intersectional strata of gender, race/ethnicity (history of migration) and/or socio-economic status (indicated by income, education, and/or occupation). Due to the diverse assessment of the intersectional strata as well as methodological aspects, comparability is limited and results are inconclusive (18, 20). For the European or German context, so far only few studies were conducted indicating intersectional inequalities in mental health status according to female gender, low income, and history of migration (20, 21).

By applying the novel approach of intersectional multilevel analysis of individual heterogeneity and discriminatory accuracy (MAIHDA) to health anxiety as an important mental health outcome, we explored the broader social context of mental health disparities with the focus on the social dimensions gender, history of migration, and income as three key social determinants of health. The following research questions were investigated: Are there social inequalities in health anxiety in the adult population living in Germany? If so, are these social inequalities of additive or multiplicative effect and which of the three social dimensions contributes most to inequalities in health anxiety? Are the more disadvantaged intersectional strata significantly greater affected by health anxiety than the more privileged ones?

## Materials and methods

### Study design and sample

Analyses were based on cross-sectional data collected via a telephone survey (computer assisted telephone interview) of the adult population (age ≥ 18 years) living in Germany. The survey was conducted from March until May 2022 by a company specialized on social research. To ensure a probability sample, randomization followed the dual-frame approach with random-digit-dialing (22) including registered and computer-generated telephone (70%) as well as mobile phone numbers (30%). Furthermore, within households, respondents were randomly chosen via the Kish selection-grid (23). Sample size calculation was based on a vignette design applied in the study. For details, please see the published study protocol (24). In the present analyses, the vignettes were not used. Interviews were conducted in German language. Accordingly, people with insufficient German language skills were excluded. Oral informed consent was given at the beginning of the interview. In total, *N* = 2,413 people participated in the study, resulting in a response rate of 45%. To correct for selection bias regarding overrepresentation or underrepresentation of certain groups of people, data was weighted following a standardized three steps approach (22, 25). First, the distribution of household sizes in the sample was weighted according to the official distribution in the population. Second, design weights were calculated to correct for differing probabilities of selection rooted in the sampling design, including the household size, number of households and mobile phone numbers. Third, continuing bias in the distribution of socio-demographic characteristics was adjusted by applying the iterative proportional fitting including age, gender, education, and place of residence. After the three-steps weighting approach the weighted study sample corresponded to the socio-demographic distribution of the official 2022 German statistics.

### Measures

#### Social dimensions

Gender, history of migration, and net household income per month were assessed by a standardized questionnaire. Since only two participants reported their gender as diverse, they were excluded from the analyses and the variable gender was dichotomized into male and female. According to the definition of migration background by the Federal Statistical Office, history of migration was assessed based on the nationality (German nationality and/or another) and country of birth of participants as well as the country of birth of both parents (born in Germany yes/no) (26). Hence, participants were allocated to the following three categories: no history of migration, parent(s) immigrated, or immigrated themselves. Due to the historical context of so called guest workers, late emigrants, and refugees, German research mostly assesses history of migration instead of race/ethnicity (14, 26). To take differences in household size and composition into account, the net monthly household income was adjusted for household size (27). The resulting equivalent income was divided into the following terciles: low (≤ 1,250 Euro), medium (1,251 – < 2,250 Euro), and high (≥ 2,250 Euro) income.

#### Covariates

Age was assessed as another item by the standardized questionnaire and divided into the following terciles: 18–40 years, 41–60 years, 60+ years. Since health anxiety can be associated with worse health conditions, it is important to account for individual health status. As an indicator of the current health status, somatic symptom severity was assessed with the German version of the Somatic Symptom Scale-8 (SSS-8) (28). The scale consists of eight items asking about gastrointestinal, cardiopulmonary, pain-related, and fatigue-related somatic symptoms, using a time interval of the last seven days. Each item can be scored on a 5-point Likert scale (0 = not bothered at all to 4 = bothered very much). The total score of the added items ranges from 0 to 32 with higher scores indicating higher somatic symptom severity (28).

#### Outcome

Health anxiety was assessed with the validated German version of the Whiteley Index-7 (WI-7), a short form of the validated Whiteley Index-14 (2). The WI-7 consists of seven items regarding illness convictions and illness worries. Each item can be answered with no (= 0) or yes (= 1). The total score ranges from 0 to 7 with higher scores indicating more health anxiety (29). In the present study, Cronbach’s *α* was 0.67.

### Missing data

In total, about 1% of individual items across all variables were missing (at random). This was mostly due to missing values on the income variable (about 18%). For the other variables, the amount of missing values was <1%. The missing data pattern was analyzed, and missing data was imputed using the multivariate imputation by chained equations method (30). The method for imputing missing values depends on the variable’s nature. For continuous variables, predictive mean matching was applied, while logistic regressions were used for binary variables.

### Statistical analyses

For an overview of the study sample in terms of health anxiety, we first calculated arithmetic mean with standard deviation (SD) of the WI-7 score. With analyses of variance, unidimensional social inequalities in health anxiety were examined. Additionally, Pearson correlation (*r*) between health anxiety and somatic symptom severity was measured.

For the intersectional analysis, MAIHDA was carried out (19, 31). This novel approach in quantitative intercategorical intersectionality research includes multilevel models nesting individuals at level 1 (between-strata level) within intersectional strata at level 2 (within-strata level). By this, social identities correspond to contextual-level variables with equal relevance for the outcome, consistent with intersectionality theory (19). In accordance with existing methods, three MAIHDA models were fitted (32). Model 1 as a base model with the intersectional strata dimensions as random intercepts; models 2a-c as partially adjusted intersectional models including each social dimension as separate fixed effect; model 3 as a fully adjusted intersectional interaction model with all three social dimensions as fixed effects. For the models, the intra-class correlation (ICC), the between-stratum variance, and the proportional change in the between-stratum variance (PCV) were calculated. The ICC describes the discriminatory accuracy of the model in identifying individuals with higher or lower health anxiety. Higher ICC indicates a greater similarity in health anxiety within the intersectional strata and a greater difference between the intersectional strata. The PCV indicates how much of the total proportion of explained variance by the intersectional strata can be contributed to each of the social dimensions. Therefore, model 2a-c and model 3 were compared to model 1. To answer the question whether social inequalities in health anxiety were of additive or multiplicative effect, the PCV (and corresponding ICC) of model 3 was assessed. A high PCV (close to 1) indicates additive effects, as the social dimensions (included as fixed effects) explain all the variance, whereas a low PCV indicates multiplicative effects beyond additive effects. To identify the social dimension contributing most to social inequalities in health anxiety, models 2a-c were compared to each other regarding the estimate (highest coefficients), the ICC (lowest coefficients), and the PCV (highest coefficients). To gain insights into the amount of health anxiety in the intersectional strata, estimated marginal means (emm) of the WI-7 score based on the random effects of model 1 were calculated. By applying post-hoc pairwise comparison, the question whether disadvantaged intersectional strata (including female gender, a history of migration, and low income) were significantly greater affected by health anxiety than the more privileged ones (including male gender, no history of migration, and high income) was pursued. We obtained 18 intersectional strata, which were derived from the three social dimensions gender, history of migration, and income (2 × 3 × 3 = 18).

To comply with model assumptions, especially to adjust for right-skewness of the WI-7, the logarithmized WI-7 score was used as outcome for all models and coefficients were back-transformed (33). All MAIHDA models were adjusted for age and somatic symptom severity. It would have been preferable to also include these variables as social dimensions. However, this was not possible due to the insufficient sample size. All analyses were carried out using weighted data to correct for selection bias and improve the accuracy and reliability of the results obtained from the data analysis. In terms of robustness, analyses using weighted and unweighted data yielded comparable results. The significance level for *p*-values was set to *p* < 0.05. Analyses were conducted in R 4.2.2 using the packages “mice” (30), “glmmTMB” (34), and “ggeffects” (35).

### Ethics approval

The survey is part of a large interdisciplinary project on persistence of somatic symptoms. The framework and study design of the Research Unit 5211 “Persistent SOMAtic Symptoms ACROSS Diseases: From Risk Factors to Modification (SOMACROSS)” and the subproject “Social Inequalities in Aggravating Factors of Persistent Somatic Symptoms (SOMA.SOC)” have been described in detail in the published study protocols (24, 36). The study was approved by the Ethics Commission of the Hamburg Medical Chamber (No. 2020-10194-BO-ff). All research was performed in accordance with relevant guidelines/regulations and the Declaration of Helsinki.

## Results

Table 1 gives an overview of the study sample in terms of health anxiety. The mean WI-7 score of the total study population was 1.69 (SD = 1.70). Significant differences in health anxiety in the two social dimensions income and history of migration as well as age (as a covariate) indicate unidimensional social inequalities in health anxiety. Regarding gender, no significant differences emerged. Somatic symptom severity was positively associated with health anxiety.

**Table 1:** Health anxiety (WI-7, range 0–7) according to social dimensions and covariates (*n* = 2,411).

	%	Mean (SD)	p*
**Total**		1.69 (1.70)	
**Social dimensions**			
**Gender**			0.458
Male	48.9	1.66 (1.59)	
Female	51.1	1.71 (1.80)	
**History of migration**			0.000
No history of migration	77.4	1.59 (1.67)	
Parent(s) immigrated	11.2	1.93 (1.80)	
Immigrated themselves	11.4	2.13 (1.88)	
**Income (in Euro)**			0.000
High (≥ 2,100)	34.5	1.34 (1.48)	
Medium (1,251 – < 2,100)	29.8	1.49 (1.56)	
Low (≤ 1,250)	35.7	2.19 (1.90)	
**Covariates**			
**Age groups**			0.000
18–40	32.2	1.39 (1.56)	
41–60	34.0	1.92 (1.81)	
≥ 61	33.8	1.74 (1.68)	
	Mean (SD)	*r* (95%-CI)	*p*
**Somatic symptom severity (SSS-8)**	5.85 (5.70)	0.55 (0.53–0.58)	0.000

CI, Confidence interval; *r,* Pearson correlation.

^*^Analyses of variance.

The intersectional MAIHDA models are presented in Table 2. According to the base model 1, social inequalities in health anxiety are apparent. The ICC was 0.039 and the conditional *R*^2^ 0.251.

**Table 2:** Results from the intersectional multilevel analysis of individual heterogeneity and discriminatory accuracy (MAIHDA) in health anxiety (WI-7; *n* = 2,411).

	Model 1 – base model^a^	Model 2a – gender adjusted^a^	Model 2b – migration adjusted^a^	Model 2c – income adjusted^a^	Model 3 – interaction effects^a^
	Estimate (95%-CI)	Estimate (95%-CI)	Estimate (95%-CI)	Estimate (95%-CI)	Estimate (95%-CI)
**Fixed effects**					
Intercept	1.56 (1.46–1.67)	1.59 (1.46–1.73)	1.46 (1.35–1.59)	1.43 (1.31–1.56)	1.44 (1.36–1.52)
**Gender** (Ref male)					
Female		0.95 (0.85–1.07)			0.91 (0.86–0.96)
**History of migration** (Ref none)					
Parent(s) immigrated			1.07 (0.95–1.21)		1.07 (0.99–1.14)
Immigrated themselves			1.15 (1.02–1.30)		1.14 (1.07–1.22)
**Income** (Ref high)					
Medium				1.07 (0.95–1.19)	1.04 (0.99–1.10)
Low				1.19 (1.07–1.33)	1.17 (1.10–1.23)
**Summary statistics**					
ICC	0.039	0.036	0.027	0.020	0.000
Between-stratum variance (SD)	0.01 (0.11)	0.01 (0.10)	0.01 (0.09)	0.01 (0.08)	0.00 (0.01)
PCV		0.083	0.331	0.495	0.995
*R*^2^_marginal/conditional_	0.221 / 0.251	0.220 / 0.248	0.231 / 0.251	0.249 / 0.246	0.253 / 0.253

CI, Confidence interval; ICC, Intra-class correlation; PCV, Proportional change in variance; SD, Standard deviation; *R*^2^_marginal_, Variance explained by fixed effects; *R*^2^_conditional_, Variance explained by fixed and random effects.

^a^Adjusted for age, somatic symptom severity (SSS-8).

The social inequalities found are most likely of additive effect. This can be concluded from the PCV of 0.995 and a corresponding ICC of 0.000 of model 3, indicating that all of the explained variance by the intersectional strata can be ascribed to the three social dimensions. Furthermore, among the three social dimensions, low income contributed most to the social inequalities found in health anxiety. This can be inferred from the highest coefficient of 1.19, the highest PCV of 0.495 and the corresponding lowest ICC of 0.020 in model 2c (partially adjusted models 2a-c).

**Figure 1:**
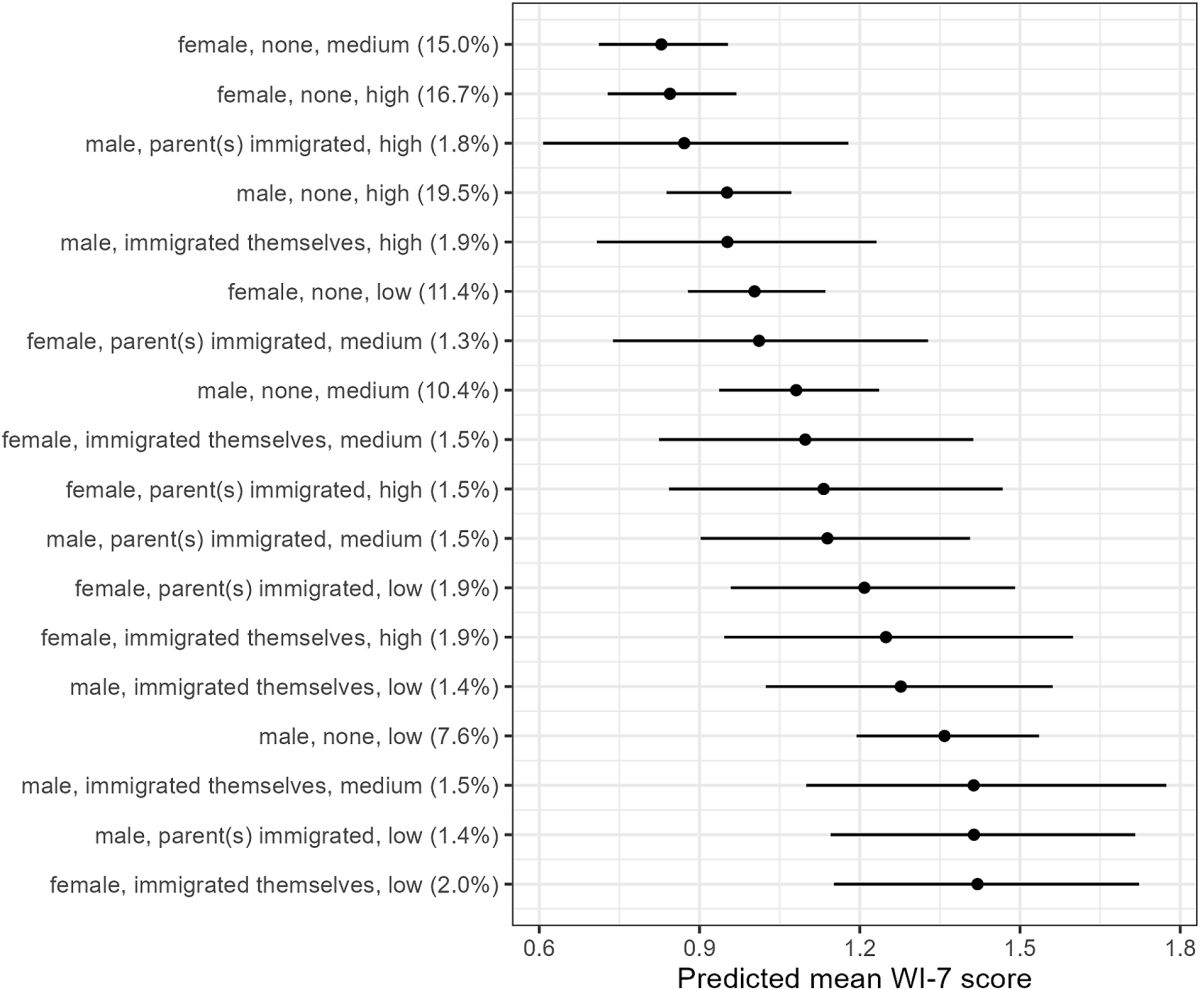
Estimated marginal means of health anxiety (WI-7) across 18 intersectional strata defined by combinations of gender, history of migration, and income based on model 1 (*n* = 2,411). Gender: male/female, history of migration: none/parent(s) immigrated/immigrated themselves, income: high/medium/low. (%) Proportion of intersectional strata in the sample. Adjusted for age, somatic symptom severity (SSS-8).

Figure 1 presents the estimated marginal means of the WI-7 score across the 18 intersectional strata derived from base model 1. The intersectional strata most affected by health anxiety were females with low income who immigrated themselves (emm = 1.42, 95%-CI 1.15–1.72), males with low income whose parent(s) immigrated (emm = 1.41, 95%-CI 1.15–1.72), and males with medium income who immigrated themselves (emm = 1.41, 95%-CI 1.10–1.77). In contrast, the intersectional strata least affected by health anxiety were females with no history of migration and medium (emm = 0.83, 95%-CI 0.71–0.95) or high income (emm = 0.84, 95%-CI 0.73–0.97), and males with high income whose parent(s) immigrated (emm = 0.87, 95%-CI 0.61–1.18). The WI-7 score of the most affected intersectional strata was about 0.6 points higher than the least affected intersectional strata. The differences between the most and least affected intersectional strata were statistically significant (*p* < 0.001). However, no clear intersectional gradient emerged.

## Discussion

### Summary of main results and discussion of current state of research

Our study contributes to the understanding of intersectional mental health disparities. By applying the novel intersectional MAIHDA, we examined the broader social context of health anxiety as an important but overlooked mental health outcome. In particular, we investigated 18 intersectional strata derived from three key social determinants of health, namely gender, history of migration, and income, which were previously examined separately, if at all. Thus, differentiating between one’s own and parent(s) immigration provides new insights into the intersections of people with a history of migration. Previously, in intersectional research, these were commonly divided into born inside or outside of the respective country (18, 20). Using intersectional MAIHDA has several advantages compared to conventional multivariate analyses, for instance the differentiation of between- and within-stratum variance and robust estimates (19, 31).

Our analyses revealed additive social inequalities in health anxiety. Among the three social dimensions, low income contributed most to these social inequalities. However, no clear intersectional gradient emerged. The intersectional strata most affected by health anxiety were females who immigrated themselves with low income, males whose parent(s) immigrated with low income, and males who immigrated themselves with medium income. On the contrary, the intersectional strata least affected by health anxiety were females with no history of migration and medium or high income, and males whose parent(s) immigrated with high income. The intersectional strata most and least affected by health anxiety differed significantly from each other.

As hypothesized, the intersection a priori set as the most disadvantaged, namely females with low income who immigrated themselves, were most affected by health anxiety. The high (mental) health burden of this disadvantaged intersectional stratum is in line with other studies relating to intersectional mental health disparities (18, 20, 21). The second intersectional strata particularly affected by health anxiety were males with low income whose parent(s) immigrated. Since the differentiation of history of migration is specific to German health research, we refer to studies investigating the German population. Concordant, a longer duration of residence was found to be an important determinant of depressive symptoms and worse subjective health status, especially among men (14, 37). Contrary to our expectations, the intersectional strata a priori defined as the most advantaged, namely males with no migration history and high income, only had the fourth lowest level of health anxiety. Rather, females with no history of migration and medium income were least affected by health anxiety. Further intersectional studies regarding health anxiety or similar mental health outcomes like generalized anxiety disorder or somatoform disorders are needed to contextualize these findings.

The WI-7 score of the most affected intersectional strata (emm = 1.42) was 0.6 points higher compared to the least affected intersectional strata (emm = 0.83). According to the validation study by Fink et al. (2) this corresponds to an increase in the WI-7category from low (0 points) to medium (1–2 points). This is relevant for healthcare, since a WI-7 of one point or higher was associated with at least one ICD-10 somatoform disorder (2). Furthermore, an increase of the WI-7 of minimum one point was associated with a significant increase in health care use and costs as well as reduced self-rated health (38).

The social inequalities in health anxiety found in our study were most likely of an additive effect. In light of the current discussion regarding multiplicative or additive intersectional effects, the latter does not disprove intersectionality in health anxiety, as intersectionality is above all a framework to understand heterogeneity and social power rather than a testable hypothesis (16).

Against the background of low income as the most impactful of the three investigated social dimensions, our study revealed further evidence for the great importance of economic deprivation in light of other social characteristics such as history of migration (14). The relevance of socioeconomic disadvantage in health anxiety was also demonstrated in a recent meta-analysis (9). The etiology of health anxiety is strongly related to negative life events, negative illness experiences, and ongoing life stressors (4). Besides structural oppression, psychosocial, material, and behavioral stressors (subsumed as intermediary determinants) as well as higher health burden and limited access to health care occur more often in the course of a low socio-economic background (as well as pre, during, and post-migration). These factors can contribute to increased health anxiety in disadvantaged intersectional strata due to concerns about individual well-being and limited individual as well as structural resources to address stress exposure and health issues appropriately (10, 14).

### Limitations

Some methodological aspects have to be considered when interpreting our results. To reduce selection bias affecting internal and external validity, a weighted data set based on a standardized three steps weighting-approach was used (22, 25). However, the study sample did not fully cover the population with a history of migration (39) because individuals with insufficient German language skills were excluded. Since history of migration showed to be a significant predictor of health anxiety, associations might be even stronger for some subgroups like refugees. Future migration-related research should cover different languages to gain access to specific migration populations like refugees (14). In addition, regarding external validity, generalizability of our results to other countries is limited due to country-specific social structures. In terms of internal validity, our cross-sectional data solely provided evidence regarding associations but no conclusions regarding causality. Hence, longitudinal data is needed to shed light on the causal relationship between the social dimensions and health anxiety, particularly in light of the feedback effect of ill health on the socioeconomic status, e.g., due to worse employment (10).

Concerning construct validity, although validated self-reported measures were used, especially in the context of telephone interviews, aspects like social desirability might have affected the measurement validity (40). Against the background of measurement invariance, future research should also focus on differences in language and cultural habits in diverse populations when implementing appropriate assessment tools (41). Besides, intersectionality served as a theoretical framework including three key social determinants of health. However, future studies could enhance the implementation of the framework in quantitative research by including further social dimensions, like age (1), gender practice and/or sexual orientation (12), and intensify the assessment of structural discrimination (42).

## Conclusion

Intersectionality serves as a framework in health disparity research to examine the complex interaction of multiple forms of disadvantage or oppression and the influence on peoples’ health experiences. By applying intersectional MAIHDA, the study revealed additive social inequalities in health anxiety as one important mental health outcome. Low income showed greatest impact, but no clear intersectional gradient emerged. Most affected by health anxiety were females who immigrated themselves with low income, males whose parent(s) immigrated with low income, and males who immigrated themselves with medium income. To reduce social and health-related inequalities in general, a more just distribution of key material and social resources is needed. Therefore, interventions should address intersectional inequalities on the structural as well as intermediary level, e.g., equalizing (gender-specific) income differences, creating a healthy working and living environment as well as diversity-friendly (access to) healthcare, and strengthening the sense of belonging in multi-cultural communities. To reduce inequalities in health anxiety in particular, proportionate universal interventions combining broad multi-language screening in outpatient and inpatient care and enabling access to targeted psychotherapeutic treatment seem promising. Future intersectional research should emphasize the actual experiences of disadvantage and privilege and apply novel approaches like MAIHDA as well as qualitative research to disaggregate the complex interaction of social categories contributing to mental health disparities.

## Data availability statement

The raw data supporting the conclusions of this article will be made available by the authors, without undue reservation.

## Ethics statement

The studies involving humans were approved by Ethics Commission of the Hamburg Medical Chamber (No. 2020-10194-BO-ff). The studies were conducted in accordance with the local legislation and institutional requirements. Written informed consent for participation was not required from the participants or the participants’ legal guardians/next of kin because data was collected via a telephone survey and oral informed consent was given at the beginning of the telephone interview.

## Author contributions

RB: Writing – original draft, Writing – review & editing, Conceptualization, Formal analysis. DL: Writing – review & editing, Methodology. OK: Writing – review & editing, Conceptualization.
